# Evaluation of the roles of the cytosolic N-terminus and His-rich loop of ZNT proteins using ZNT2 and ZNT3 chimeric mutants

**DOI:** 10.1038/s41598-018-32372-8

**Published:** 2018-09-20

**Authors:** Kazuhisa Fukue, Naoya Itsumura, Natsuko Tsuji, Katsutoshi Nishino, Masaya Nagao, Hiroshi Narita, Taiho Kambe

**Affiliations:** 1 Division of Integrated Life Science, Graduate School of Biostudies, Kyoto University, Kyoto, 606-8502 Japan; 2 Department of Food Science, Kyoto Women’s University, Kyoto, 605-8501 Japan

## Abstract

The physiological roles of Zn transporter (ZNT) proteins are being increasingly recognized, and three dimensional structures of ZNT bacterial homologs have facilitated our understanding of their biochemical characteristics at the molecular level. However, the biological role of the unique structural features of vertebrate ZNTs, which are absent in their bacterial homologues, is not completely understood. These ZNT sequences include a cytosolic His-rich loop between transmembrane helices IV and V and the cytosolic N-terminus. This study investigated the contribution of these features to zinc transport by ZNT proteins. The importance of the His residues in the cytosolic His-rich loop was investigated using ZNT2 Ala substitution and deletion mutants. The presence of His residues was not essential for zinc transport, even though they possibly participate in modulation of zinc transport activity. Furthermore, we determined the role of the N-terminus by characterizing ZNT2 and ZNT3 domain-swapped and deletion mutants. Unexpectedly, the N-terminus was also not essential for zinc transport by ZNT2 and the domain-swapped ZNT2 mutant, in which the cytosolic His-rich loop was substituted with that of ZNT3. These results provide molecular insights into understanding the roles of the cytosolic parts of ZNT2, ZNT3, and probably other members of their subgroup.

## Introduction

Zn transporter (ZNT) proteins encoded by the *SLC30A* group of genes are indispensable zinc transporters, which sequestrate cytosolic zinc into intracellular compartments or efflux zinc to the extracellular space^1–5^. ZNTs play pivotal roles in human physiology. Recently, single nucleotide polymorphisms (SNPs) in *SLC30A* genes have been shown to be associated with several inherited disorders. SNPs in *ZNT8/SLC30A8* and *ZNT3/SLC30A3* are associated with the risk of developing type-2 diabetes mellitus^6–9^ and gender-specific schizophrenia^10^, respectively. In addition, mutations in *ZNT2/SLC30A2* result in transient neonatal zinc deficiency (TNZD) in breastfeeding infants of affected mothers^11–13^, whereas *ZNT10/SLC30A10* mutations cause Parkinsonism and dystonia with hypermagnesemia, polycythaemia, and hepatic cirrhosis^14,15^. These results indicate that molecular studies on ZNTs are important for understanding their pathophysiological and biochemical properties.

**Figure 1:**
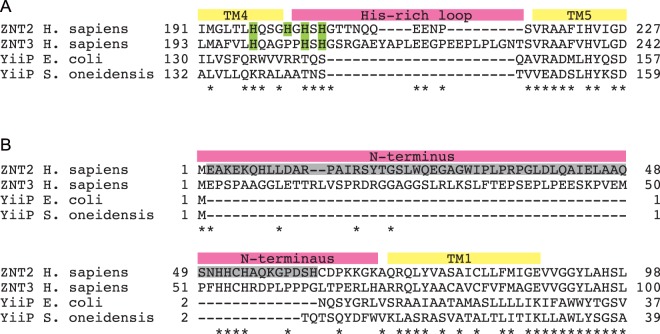
Alignment of ZNT2 and ZNT3 amino acid sequences of the cytosolic His-rich loop and the cytosolic N-terminus. (**A**) Alignment of the cytosolic His-rich loop of human ZNT2 (residues 191–227) and ZNT3 (residues 193–242). Predicted TM helices IV and V (based on YiiP^16^) are labelled and His residues of the His-rich loop are highlighted in green. (**B**) Alignment of human ZNT2 (residues 1–98) and ZNT3 (residues 1–100) N-terminal sequences preceding the first TM helix. The sequence (Glu2 to His62) of ZNT2 deleted is shaded in gray. In (**A**) and (**B**), sequences of *E*. *coli* and *S*. *oneidensis* YiiP were also aligned for comparison. Amino acids identical between ZNT2 and ZNT3 sequences are indicated by *.

Three-dimensional (3D) structures of YiiP, the *Escherichia coli* and *Shewanella oneidensis* ZNT homologue, obtained using X-ray crystallography and cryo-electron microscopy, have enhanced our understanding of the structural and biochemical properties of ZNTs^16–20^. YiiP forms homodimers with six transmembrane (TM) helices and functions as a proton-zinc exchanger. Most ZNTs form similar homodimers with six TM helices for transporting zinc across biological membranes^21–25^ and functioning as proton-zinc exchangers^26,27^. However, some ZNTs, including ZNT5 and ZNT6, also form heterodimers^22,23,28,29^, and ZNT5 forms 15 TM helices. Despite valuable insights from YiiP structure, the structural and biochemical features of ZNTs have not been completely characterized because of several unique features of ZNT sequences that are not present in YiiP. The ZNT-specific features include the cytosolic His-rich loop between TM helices IV and V and the sequence of the N-terminus (Fig. 1)^30^. Previous studies on ZNT and their plant homologues indicated that the His-rich loop might participate in zinc transport by coordinating zinc via His residues^31–35^, although the importance of His residues remains unclear. Based on the results of deletion studies, the N-terminus was also thought to be associated with zinc transport^36,37^; however, its biological function requires further investigation as results obtained using the short YiiP N-terminus cannot be used to form any tenable hypotheses.

ZNTs are subdivided into four subgroups: (1) ZnT1 and ZnT10, (2) ZnT2, ZnT3, ZnT4, and ZnT8, (3) ZnT5 and ZnT7, and (4) ZnT6^2,3,5^ (hereafter, these subgroups will be referred to as ZNT subgroup I, II, III, or IV). Previously, we biochemically characterized the members of ZNT subgroups I and III using their domain-swapped and deletion mutants. Specifically, we directly compared the properties of ZNT1 and ZNT10 or of ZNT5 and ZNT7 using genetically engineered DT40 cells^22,38–40^. In this study, we investigated the biochemical properties of ZNT subgroup II members, ZNT2 and ZNT3, as the zinc transport functions of wild-type (WT) ZNT2 and zinc-transport competent mutants can be easily evaluated by expressing these proteins in *znt1*^*−/−*^*mt*^*−/−*^*znt4*^*−/−*^ cells; furthermore, cellular zinc resistance in high zinc culture conditions and protein expression level can be monitored^24,38,41,42^, and the observations can be compared with those of cells expressing ZNT3, which shows low zinc transport activity despite high sequence similarity with ZNT2^24,30^. Our results enhance our understanding of the biochemical characteristics of ZNT subgroup II members and ZNTs in general.

## Results

### His residues of the ZNT2 cytosolic His-rich loop

**Figure 2:**
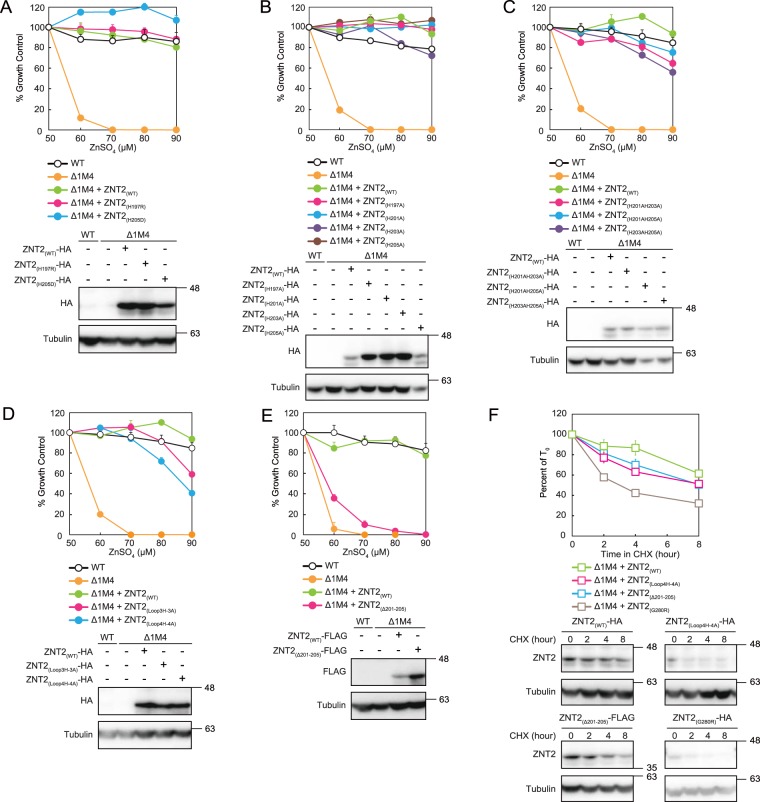
His residues of the cytosolic ZNT2 His-rich loop are not essential for zinc transport. (**A**) Zinc transport activity of *znt1*^*−/−*^*mt*^*−/−*^*znt4*^*−/−*^ cells expressing H197R or H205D ZNT2 SNP mutants (ZNT2_(H197R)_, ZNT2_(H205D)_) was comparable to that of WT ZNT2-expressing cells. (**B**) Expressing ZNT2 mutants with single His to Ala substitution in the His-rich loop (ZNT2_(H197A)_, ZNT2_(H201A)_, ZNT2_(H203A)_, and ZNT2_(H205A)_) in *znt1*^*−/−*^*mt*^*−/−*^*znt4*^*−/−*^ cells resulted in zinc transport activity comparable to that of WT ZNT2. Similar observations were made with the double substitution mutants (ZNT2_(H201AH203A)_, ZNT2_(H201AH205A)_, and ZNT2_(H203AH205A)_) (**C**), whereas expression of ZNT2 mutants with three or four Ala-substituted His loop residues (ZNT2_(Loop3H-3A)_ and ZNT2_(Loop4H-4A)_), moderately decreased zinc resistance (**D**). (**E**) Expression of ZNT2 mutant with the deleted cytosolic His-rich loop (ZNT2_(Δ201-205)_) in *znt1*^*−/−*^*mt*^*−/−*^*znt4*^*−/−*^ cells did not confer zinc resistance. In experiments depicted in panels (**A**–**E**), cells were grown in the presence of indicated concentrations of ZnSO_4_ and the number of surviving cells was estimated using the alamarBlue assay in triplicate (representative results shown). Expression of WT or mutant ZNT2 proteins in *znt1*^*−/−*^*mt*^*−/−*^*znt4*^*−/−*^ cells was confirmed by immunoblotting (lower sub-panels). Tubulin was used as the loading control. (**F**) Comparison of the stability of ZNT2_(Loop4H-4A)_ and ZNT2_(Δ201-205)_ mutants with those of WT ZNT2 or ZNT2_(G280R)_ mutant. The expression levels of each protein at each time point are shown, with representative results of immunoblotting depicted in the lower panel. The results of ZNT2_(G280R)_ is shown as a control for the destabilized ZNT2 mutant^42^. Data show mean ± SEM of triplicate experiments (lower sub-panels). Tubulin was used as the loading control.

We have previously reported that *znt1*^*−/−*^*mt*^*−/−*^*znt4*^*−/−*^ cells stably expressing the known H205D ZNT2 mutant (listed in the dbSNP database) showed zinc resistance similar to that of cells expressing WT ZNT2^40^. This was unexpected as His 205 is in the cytosolic His-rich loop between TM helices IV and V (Fig. 1A), which is considered important for zinc transport activity of ZNTs^3,4^. Hence, we examined another dbSNP ZNT2 mutant, H197R, in *znt1*^*−/−*^*mt*^*−/−*^*znt4*^*−/−*^ cells; similar to the H205D mutant, no defects in zinc transportation were observed (Fig. 2A) (Supplementary Table 1).

These results prompted us to investigate the importance of loop His residues using Ala scanning. The His-rich loop of ZNT2 contains four His residues; however, H197 may belong to the TM helix IV. When stably expressed in *znt1*^*−/−*^*mt*^*−/−*^*znt4*^*−/−*^ cells, monosubstituted H197A, H201A, H203A, and H205A mutants showed zinc transport similar to that of WT ZNT2 and H197R and H205D mutants (Fig. 2B). These observations indicate that a specific His residue possibly does not control zinc transport. Similar results were obtained with double Ala mutants ZNT2_(H201AH203A)_, ZNT2_(H201AH205A)_, and ZNT2_(H203AH205A)_ (Fig. 2C), suggesting that substitution of two His residues in the loop with Ala does not affect zinc transport activity. Furthermore, cells expressing triple (ZNT2_(Loop3H-3A)_ with Ala substituting H201, H203, and H205) and quadruple Ala mutants (all loop His residues substituted; ZNT2_(Loop4H-4A)_) still showed (moderately decreased) zinc-resistance (Fig. 2D).

As deletion of the loop segment containing the His residues (His-rich cluster) leads to loss of zinc transport activity of cation diffusion facilitator (CDF) transporters, including ZNTs^31,32^, we designed a deletion mutant of ZNT2, in which the loop segment between H201 and H205 residues was deleted (ZNT2_(Δ201-205)_), and evaluated zinc resistance of cells expressing this mutant. Compared to the resistance of cells expressing ZNT2_(Loop3H-3A)_, the stable expression of ZNT2_(Δ201-205)_ in *znt1*^*−/−*^*mt*^*−/−*^*znt4*^*−/−*^ cells significantly decreased zinc resistance (Fig. 2E). Next, we assessed the differences in stability between ZNT2_(Δ201-205)_ and ZNT2_(Loop4H-4A)_ for investigating the importance of these His residues in detail. Cells expressing each mutant were treated with cycloheximide to block further protein synthesis, and the protein expression levels were monitored periodically over 8 h by immunoblotting (Fig. 2F). Compared to WT ZNT2, the stability of both proteins decreased slightly, but they were more stable than the ZNT2_G280R_ mutant, which is known to be a TNZD-causing unstable mutant with impaired zinc transport activity^42^. These results suggest that His residues of the His-rich ZNT2 loop are not required for zinc transport. However, these residues possibly participate in zinc coordination during zinc transport, as zinc resistance was moderately reduced in ZNT2_(Loop3H-3A)_ and ZNT2_(Loop4H-4A)_ mutants.

In addition, we constructed 15 ZNT2 SNP mutants (Supplementary Table 1), among which E279K displayed reduced zinc transport and protein stability (Supplementary Fig. 1A,B). This result is interesting, as Glu279 is hypothesized to form intermolecular salt-bridges in ZNT2, analogous to the salt bridges observed in YiiP structures^17,18^, and may provide useful information for diagnosing TNZD (see Discussion).

### Biochemical properties of domain-swapped ZNT2 and ZNT3

**Figure 3:**
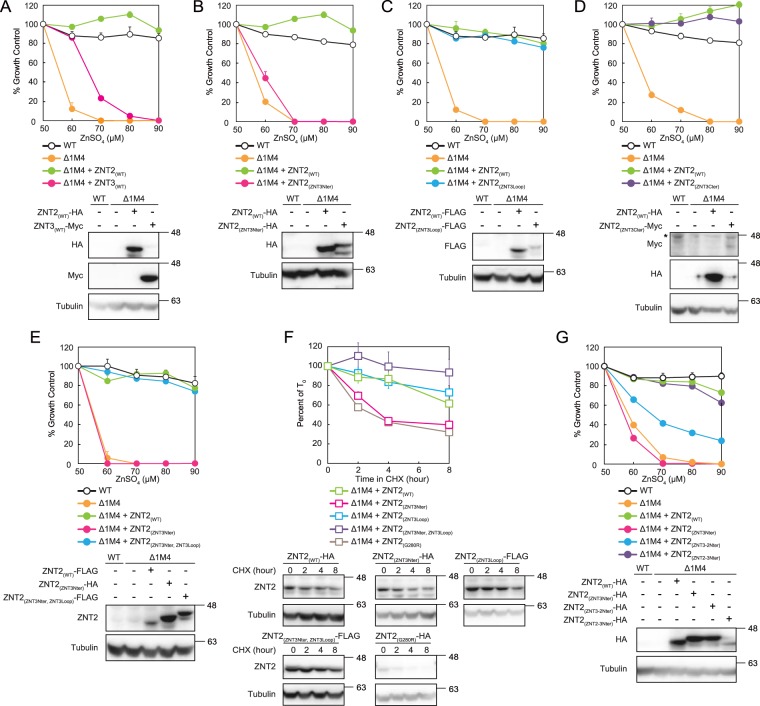
Domain swapping analysis of ZNT2 and ZNT3 on the ZNT2 backbone. (**A**) Expression of ZNT3 in *znt1*^*−/−*^*mt*^*−/−*^*znt4*^*−/−*^ cells did not confer significant resistance to high zinc concentrations, compared to the zinc resistance of cells expressing ZNT2. (**B–D**) Expression of ZNT2_(ZNT3Nter)_ in *znt1*^*−/−*^*mt*^*−/−*^*znt4*^*−/−*^ cells did not confer zinc resistance, whereas expression of ZNT2_(ZNT3Loop)_ and ZNT2_(ZNT3Cter)_ conferred zinc resistance, which was similar to that of WT ZNT2-expressing cells. In (**D**) *indicates the position of non-specific band. (**E**) Expression of ZNT2_(ZNT3Nter__,__ZNT3Loop)_ conferred zinc resistance in *znt1*^*−/−*^*mt*^*−/−*^*znt4*^*−/−*^ cells. (**F**) Stabilities of ZNT2_(ZNT3Loop)_, ZNT2_(ZNT3Nter)_, and ZNT2_(ZNT3Nter__,__ZNT3Loop)_ were evaluated as described in Fig. 2F. Data show mean ± SEM of triplicate experiments (lower sub-panels). Tubulin was used as the loading control. (**G**) Cells expressing ZNT2_(ZNT2-3Nter)_ showed zinc resistance similar to that of ZNT2-expressing cells, whereas cells expressing ZNT2_(ZNT3-2Nter)_ showed moderate resistance. In (**A**−**E**) and (**G**), the alamarBlue assay was performed as shown in Fig. 2. Stable expression of WT and mutant ZNT2 in *znt1*^*−/−*^*mt*^*−/−*^*znt4*^*−/−*^ cells was confirmed by immunoblotting (lower sub-panels). Tubulin was used as the loading control.

ZNT3 and ZNT2 share the highest homology (56% sequence identity and 69% residues with positive alignment score, according to BLASTP). Intriguingly, in contrast to ZNT2, stable expression of ZNT3 in *znt1*^*−/−*^*mt*^*−/−*^*znt4*^*−/−*^ cells conferred limited zinc resistance; *znt1*^*−/−*^*mt*^*−/−*^*znt4*^*−/−*^ cells stably expressing ZNT3 demonstrated partial resistance to treatment with ≤70 µM ZnSO_4_ and did not grow in the presence of 80 µM ZnSO_4_ (Fig. 3A). We next attempted to characterize biochemical features of ZNTs, particularly those of the ZNT subgroup II, based on the differences between these two proteins. We constructed ZNT2 and ZNT3 domain-swapped mutants, ZNT2_(ZNT3Nter)_, ZNT2_(ZNT3Loop)_, and ZNT2_(ZNT3Cter)_, in which the ZNT3 N-terminus, His-rich loop, or the C-terminus, respectively, were grafted on the ZNT2 scaffold (Fig. 3B–D). Unexpectedly, expression of the ZNT2_(ZNT3Loop)_ did not disrupt zinc transport in *znt1*^*−/−*^*mt*^*−/−*^*znt4*^*−/−*^ cells (Fig. 3C) despite the low sequence similarity between ZNT2 and ZNT3 loops and the presence of six unique Pro residues in the ZNT3 loop (Fig. 1A). Expression of ZNT2_(ZNT3Cter)_ did not disrupt zinc transport in *znt1*^*−/−*^*mt*^*−/−*^*znt4*^*−/−*^ cells (Fig. 3D). In contrast, expression of ZNT2_(ZNT3Nter)_, in which the N-terminal sequence of ZNT2 (Met1 to Cys78) was replaced with the ZNT3 N-terminus (Met1 to Ser76) (Fig. 1B), did not confer zinc resistance (Fig. 3B). This was unexpected as we have previously observed that grafting of the N-terminal half of ZNT5 on ZNT7 did not impair ZNT7 activity^22^. The ZNT2_(ZNT3Nter)_ mutant was significantly less stable than WT ZNT2 (Fig. 3F), suggesting that the swapping of the ZNT3 N-terminus led to loss of both zinc transportation and stability of ZNT2.

Interestingly, expression of the ZNT2 mutant ZNT2_(ZNT3Nter,__ZNT3Loop)_, in which both the N-terminus and the cytosolic His-rich loop of ZNT2 are substituted with the corresponding segments of ZNT3, in *znt1*^*−/−*^*mt*^*−/−*^*znt4*^*−/−*^ cells resulted in zinc resistance comparable to that of WT ZNT2 (Fig. 3E). The stability of ZNT2_(ZNT3Nter__,__ZNT3Loop)_ was higher than that of WT ZNT2 (Fig. 3F). This observation and the above results strongly suggest that the ZNT3 N-terminus impairs zinc transport by ZNT2 and reduces its stability by perturbing the cytosolic His-rich loop of ZNT2; association with the ZNT3 N-terminus and ZNT3 His-rich loop reverses these defects.

We constructed ZNT2_(ZNT3-2Nter)_ and ZNT2_(ZNT2-3Nter)_ plasmids to further explore this hypothesis; in the former, the ZNT2 N-terminus (Met1 to Asn50) was replaced with the ZNT3 N-terminal sequence (Met1 to Phe52), whereas in the latter, the ZNT2 sequence (His51 to Cys78) was substituted with the corresponding ZNT3 sequence (His53 to Ser76) (Fig. 1B). Cells expressing ZNT2_(ZNT2-3Nter)_ showed zinc resistance comparable to that of cells expressing WT ZNT2, whereas the zinc resistance of cells expressing ZNT2_(ZNT3-2Nter)_ was moderately reduced (Fig. 3G). These results suggest that the ZNT3 N-terminal sequence (Met1 to Phe52) may be involved in impairing ZNT2 zinc transport.

**Figure 4:**
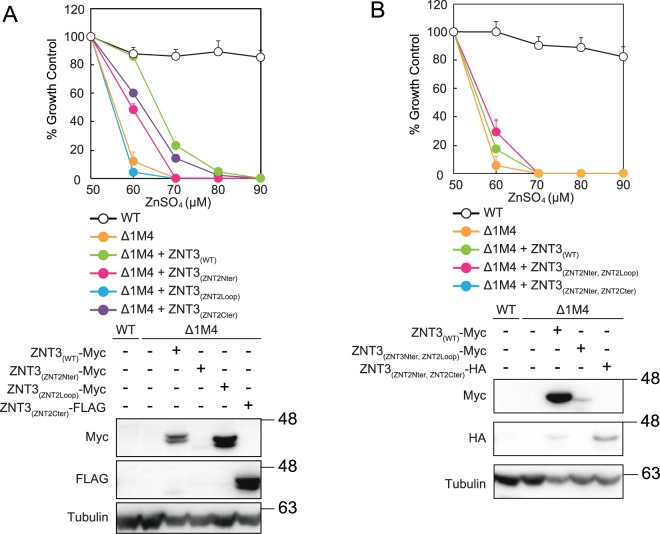
Domain swapping analysis of ZNT2 and ZNT3 on the ZNT3 backbone. (**A**) Expression of ZNT3_(ZNT2Nter)_, ZNT3_(ZNT2Loop)_, or ZNT3_(ZNT2Cter)_ in *znt1*^*−/−*^*mt*^*−/−*^*znt4*^*−/−*^ cells did not result in zinc resistance. (**B**) Cells expressing ZNT3_(ZNT2Nter__,__ZNT2Loop)_ or ZNT3_(ZNT2Nter__,__ZNT2Cter)_, with two grafted cytosolic segments, showed almost no resistance to high zinc concentrations. In (**A**,**B**), alamarBlue assay was performed as shown in Fig. 2. Stable expression of WT and mutant ZNT2 in *znt1*^*−/−*^*mt*^*−/−*^*znt4*^*−/−*^ cells was confirmed by immunoblotting (lower sub-panels). Tubulin was used as the loading control.

We constructed analogous domain-swapped ZNT2 and ZNT3 mutants based on the ZNT3 backbone: ZNT3_(ZNT2Nter)_, ZNT3_(ZNT2Loop)_, and ZNT3_(ZNT2Cter)_. However, *znt1*^*−/−*^*mt*^*−/−*^*znt4*^*−/−*^ cells expressing these mutants did not confer zinc resistance (Fig. 4A). Moreover, zinc resistance was not observed in *znt1*^*−/−*^*mt*^*−/−*^*znt4*^*−/−*^ cells expressing ZNT3_(ZNT2Nter__,__ZNT2Loop)_, and ZNT3_(ZNT2Nter__,__ZNT2Cter)_ (Fig. 4B). These observations indicate that ZNT2 structural features did not improve the innately weak zinc transportation ability of the ZNT3 backbone (TM helices) in *znt1*^*−/−*^*mt*^*−/−*^*znt4*^*−/−*^ cells (Fig. 3A).

### Analysis of the importance of the ZNT N-terminus using deletion mutants

**Figure 5:**
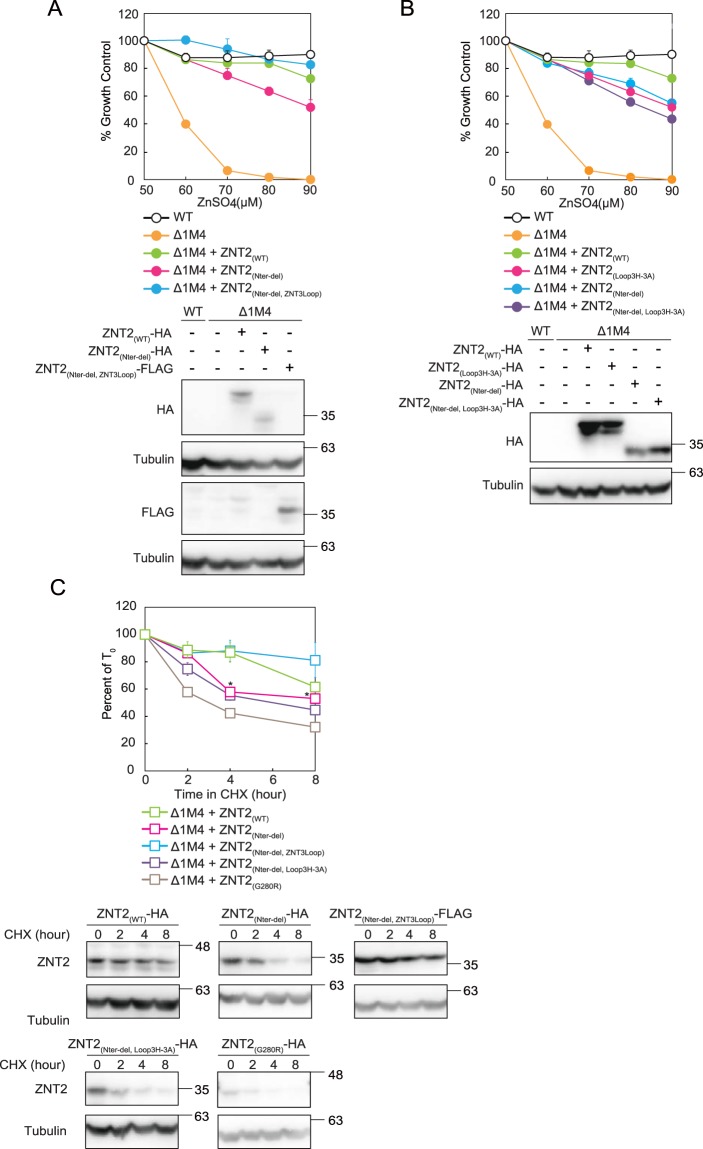
Deletion of the ZNT2 cytosolic N-terminus did not result in loss of zinc transport function. (**A**) Expression of ZNT2_(Nter-del)_ and ZNT2_(Nter-del__,__ZNT3Loop)_ in *znt1*^*−/−*^*mt*^*−/−*^*znt4*^*−/−*^ cells conferred zinc resistance, similar to (with minor loss) the effect of ZNT2_(Nter-del__,__Loop3H-3A)_ expression (**B)**. In (**A**,**B**), the alamarBlue assay was performed as shown in Fig. 2. Stable expression of WT and mutant ZNT2 in *znt1*^*−/−*^*mt*^*−/−*^*znt4*^*−/−*^ cells was confirmed by immunoblotting (lower sub-panels). Tubulin was used as the loading control. (**C**) The stabilities of ZNT2_(Nter-del)_, ZNT2_(Nter-del__,__ZNT3Loop)_, and ZNT2_(Nter-del__,__Loop3H-3A)_ were evaluated as described in Fig. 2F. Data show mean ± SEM of triplicate experiments (lower sub-panels). Tubulin was used as the loading control. Asterisk (*) denotes a significant difference between WT ZNT2, and ZNT2_(Nter-del)_ mutant protein levels (*P* < 0.05 by Dunnett’s test).

To further investigate the involvement of the N-terminus of ZNT subgroup II in zinc transportation, we constructed the ZNT2 N-terminus (Glu2 to His62) deletion mutant (ZNT2_(Nter-del)_) based on the sequences of the short N-termini of homologous *E*. *coli* and *S*. *oneidensis* YiiP proteins (Fig. 1B)^16,18^. Unexpectedly, *znt1*^*−/−*^*mt*^*−/−*^*znt4*^*−/−*^ cells expressing ZNT2_(Nter-del)_ showed zinc resistance similar to that of cells expressing WT ZNT2 (Fig. 5A). Minor contribution of the ZNT2 N-terminus to zinc transport was confirmed using the ZNT2_(Nter-del__,__Loop3H-3A)_ mutant, in which His 201, 203, and 205 residues of the cytosolic His-rich loop (see Fig. 2D) were replaced by Ala, and the N-terminus was deleted (Fig. 5B). Cells expressing ZNT2_(Nter-del__,__Loop3H-3A)_ showed zinc resistance similar to that of cells expressing ZNT2_(Nter-del)_. This observation was additionally verified by constructing ZNT2_(Nter-del__,__ZNT3Loop)_, in which the cytosolic His-rich loop of ZNT2_(Nter-del)_ was substituted by the corresponding ZNT3 sequence. Expression of ZNT2_(Nter-del__,__ZNT3Loop)_ in *znt1*^*−/−*^*mt*^*−/−*^*znt4*^*−/−*^ cells resulted in zinc resistance similar to that of cells expressing ZNT2_(Nter-del)_ (Fig. 5A). These results indicate that the N-terminus itself is not required for ZNT2 zinc transport, even in the ZNT2 mutant in which the His-rich loop is substituted with that of ZNT3. The stability of these N-terminal deletion mutants was investigated in detail. The ZNT2_(Nter-del__,__ZNT3Loop)_ mutant was more stable, whereas the ZNT2_(Nter-del)_ and ZNT2_(Nter-del__,__Loop3H-3A)_ mutants were less stable than WT ZNT2 (Fig. 5C). As the stability of ZNT2_(Nter-del__,__Loop3H-3A)_ was comparable to that of ZNT2_(Nter-del)_, the replacement of three His residues with Ala did not affect the stability of the ZNT2_(Nter-del)_ mutant.

Taken together, our results suggest that the ZNT3 N-terminus is incompatible with ZNT2 structure and perturbs its zinc transportation ability and stability; however, zinc transporting ability can be restored by combining the N-terminus and cytosolic His-rich loop of ZNT3, even when grafted on the ZNT2 backbone (TM helices).

## Discussion

**Figure 6:**
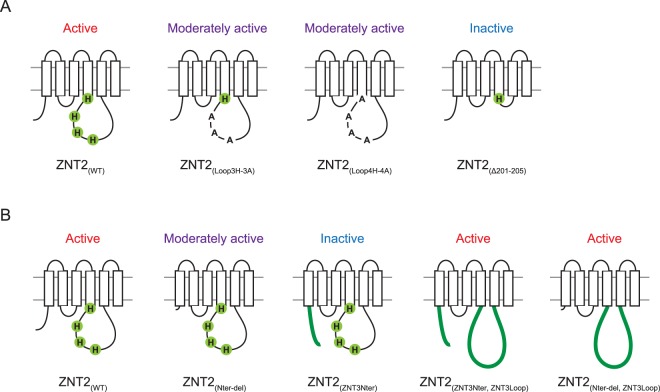
Schematic representation of the main findings of this study. His residues of the cytosolic His-rich loop (**A**) and the N-terminus of ZNTs (**B**) are not essential but are required for ZNT-mediated zinc transport. Zinc transport activities are shown above the schematic models of each construct.

In this study, we obtained two unexpected but important results regarding the molecular characteristics of ZNTs, which are summarized in Table 1. First, the His residues of the cytosolic His-rich loop are not essential for zinc transport by ZNTs, although these residues possibly participate in modulating zinc transport activity (Fig. 6A). Second, the ZNT N-terminus alone is not essential for zinc transport (Fig. 6B). Although these results were obtained from studies on the members of ZNT subgroup II, to our knowledge, this is the first report presenting evidence for these conclusions, which contribute to our understanding of ZNTs at the molecular level.

**Table 1:** Zinc resistance conferred to *znt1*^*−/−*^*mt*^*−/−*^*znt4*^*−/−*^ cells by ZNT2, ZNT3, and their representative mutants.

Expressed protein	Zinc resistance in *znt1*^*−/−*^*mt*^*−/−*^*znt4*^*−/−*^ cells
ZNT2	+ + + +
ZNT2_(Loop3H-3A)_	+ + +
ZNT2_(Loop4H-4A)_	+ +
ZNT2_(Δ201-205)_	−*
ZNT3	−*
ZNT2_(ZNT3Nter)_	−
ZNT2_(ZNT3Loop)_	+ + + +
ZNT2_(ZNT3Cter)_	+ + + +
ZNT2_(ZNT3-2Nter)_	+ +
ZNT2_(ZNT2-3Nter)_	+ + + +
ZNT2_(ZNT3Nter_, _ZNT3Loop)_	+ + + +
ZNT2_(Nter-del)_	+++
ZNT2_(Nter-del_, _ZNT3Loop)_	++++
ZNT2_(Nter-del_, _Loop3H-3A)_	+++

*Marginal cell growth was observed at 70 μM ZnSO_4_.

Relative values shown are estimates of representative results shown in Figs 2, 3, and 5 as follows: + + + + , >80% viability compared to WT ZNT2-expressing *znt1*^*−/−*^*mt*^*−/−*^*znt4*^*−/−*^ cells at 90 μM ZnSO_4_; + + + , + + , and + , reduced growth (50–80%, 20–50% and ≤20% growth relative to the viability of the corresponding WT, respectively); −no growth.

This is the first study to evaluate the importance of His residues of the cytosolic His-rich loop of ZNTs. His residues of the loop are important for zinc transportation, as this function is disrupted in mutant ZNTs and homologues lacking the His-rich cluster in the loop^31,32^. However, complete substitution analysis of this region, similar to that performed on ZIP4 proteins^43^, is required. Our observations that zinc resistance was moderately reduced in cells expressing ZNT2_(Loop3H-3A)_ and ZNT2_(Loop4H-4A)_ mutants indicate that His residues are not required for ZNT2 zinc transport, although they do participate in zinc transport. Previous studies suggest that His residues possibly coordinate zinc ions during transport or modulate substrate metal specificity^34,35^. Based on the observations that the stabilities of ZNT2_(Loop4H-4A)_ and ZNT2_(Δ201-205)_ mutants were not significantly altered, and that the ZNT2_(Δ201-205)_ mutant possesses negligible zinc resistance, we suggest that the spatial configuration of the loop is possibly more important than the presence of His residues for movement of the TM helices during zinc transport. Further studies on the role of loop sequence length are required.

The molecular functions of the ZNT N-terminus have not been as extensively investigated as those of the cytosolic C-terminus^44,45^. The homologous *E*. *coli* and *S*. *oneidensis* YiiP proteins possess short N-termini with low sequence similarity^16,18^, and diverse N-termini sequences have been observed among ZNTs. ZNT1 and ZNT10 (ZNT subgroup I) feature short N-termini, similar to YiiP^30^, while the N-terminus of the nine ZNT5 TM helices is uniquely long^46^, which renders speculation regarding N-terminus function based on sequence conservation difficult. The N-terminus was previously considered crucial for zinc transport, based on studies on complete and partial deletion ZNT2 mutants and plant homologous proteins with no zinc transport activity^36,37^. Unlike these deletion mutants, the N-terminus deletion mutants used in this study were designed based on YiiP proteins, which harbour N-termini of lengths similar to the N-termini of ZNT1 and ZNT10 (ZNT subgroup I). We show that these mutants retain zinc transport functions. These results suggest that a long N-terminal sequence is not required for forming a zinc transport-competent ZNT conformation, and that a short N-terminus is sufficient to form a compact four-helix bundle consisting of TM helices I, II, IV, and V, as observed in YiiP structures^16–20^. A recent report shows that a short ZNT8 isoform with moderate-length N-terminus retains zinc transport activity^47^, which may support our observations.

What is the role of the N-terminus in ZNT subgroup II members? Important features and sequence motifs have been previously identified in the N-termini of the members of this subfamily and other homologues, offering insights into the potential roles of this structural feature. The ZNT2 N-terminus features a His cluster motif involved in mitochondrial sorting^36^, the ZNT3 N-terminus contains a zinc binding (sensor) motif^33^, and the ZNT4 N-terminus possesses four consecutive L-(x)6 repeats resembling the Leu zipper motif for potential protein-protein interactions^48^, whereas the *At*MTP1 N-terminus contains two Cys residues, which possibly participate in zinc transport^37^. In this study, we showed that substitution of the ZNT2 N-terminus with that of ZNT3 (ZNT2_(ZNT3Nter)_) results in loss of zinc transport function of WT ZNT2, but not of the ZNT2 mutant (ZNT2_(ZNT3Nter__,__ZNT3Loop)_), the His-rich loop of which was also substituted with the corresponding ZNT3 loop. On the contrary, the ZNT2 N-terminus probably has the ability to stabilize ZNT2, as ZNT2_(Nter-del)_ was slightly less stable than WT ZNT2 (See Fig. 5C). This hypothesis was supported by the fact that ZNT2_(Nter-del,__ZNT3Loop)_ was less stable than ZNT2_(ZNT3Nter__,__ZNT3Loop)_ (See Figs 3F and 5C). These observations suggest that the N-termini of ZNT subgroup II members interact with the cytosolic His-rich loop and modulate zinc transport, which may be potentiated by proper combinations of each protein. In ZNT3, this function may be attributed to the sequence spanning Met1 to Phe52, as substituting the sequence between Met1 to Asn50 of ZNT2 with the sequence encompassing Met1 to Phe52 of ZNT3 (ZNT2_(ZNT3-2Nter)_) moderately decreased zinc transportation by ZNT2 (Table 1). Further studies are required to extend this conclusion to other ZNTs, such as members of ZNT subgroup III and IV.

Resistance of ZNT3-expressing *znt1*^*−/−*^*mt*^*−/−*^*znt4*^*−/−*^ cells to high zinc concentrations is lower than that of cells expressing ZNT2. Similar observations have been previously reported; Unlike ZNT2, which shows potent zinc transport activity in many cell lines^49–52^, ZNT3 shows weak zinc transport activity in non-neuronal cells^49^. ZNT3 is a functional zinc transporter in synaptic vesicles, where it uses specific ions as the driving force for zinc transportation^53,54^. Our results indicate that the three ZNT3 cytosolic portions (the N-terminus, His-rich loop, and C-terminus) are functional when fused to the ZNT2 backbone, although the N-terminus has to be fused simultaneously with the His-rich loop, suggesting that the TM helices, and not the cytosolic parts of ZNT3, determine ZNT3-specific zinc transport in synaptic vesicles.

Another important aspect of this study was the characterization of ZNT2 SNP mutants, as even heterozygous ZNT2 mutations may reduce zinc levels in the breast milk of affected mothers, leading to TNZD in exclusively breastfed infants^24,25,42,52,55–58^. TNZD-associated zinc deficiency is effectively counteracted by zinc supplements^12,13^ and accumulating genetic information associated with TNZD pathogenesis assists in supporting normal growth and development of breastfed infants^13,58^. Previously, we identified four ZNT2 SNPs associated with high-TNZD risk^42^, one of which was recently confirmed to be clinically relevant^59^. In this study, we report E279K as a new ZNT2 SNP associated with high-risk of TNZD, based on similarities with TNZD-causing G280R SNP (Supplementary Fig. 1A,B)^42^. Sequence-wise, the ZNT2 Glu279 residue corresponds to Asp207 and 209 residues of *E*. *coli*^16^ and *S*. *oneidensis* YiiP^18^ proteins, respectively, which form intermolecular salt-bridges with Lys77 and Lys79 YiiP residues (in *E*. *coli* and *S*. *oneidensis*, respectively), ensuring proper orientation of TM helices III and VI at the dimer interface^17^. These Lys residues correspond to the Arg138 residue in ZNT2 (see Supplementary Fig. 1C), suggesting that Glu279 possibly participates in the formation of similar salt-bridges in ZNT2.

In conclusion, our observations revealed that sequences unique to vertebrate ZNTs, including the cytosolic His-rich loop between TM helices IV and V and the cytosolic N-terminus, both of which have been previously associated with zinc transport, are not essential for zinc transport, at least by ZNT subgroup II members. Recent evidence regarding the diverse physiological functions of ZNTs and the role of impaired ZNT function in human diseases indicates that ZNTs can be developed as novel therapeutic targets. Biochemical characterization of ZNTs is important considering the growing interest in the pathophysiological functions of these proteins.

## Methods

### Cell culture, plasmid construction, and stable transfection

Chicken B lymphocyte-derived WT DT40 cells and *znt1*^*−/−*^*mt*^*−/−*^*znt4*^*−/−*^ cells were maintained in Roswell Park Memorial Institute (RPMI) 1640 medium (Nacalai Tesque, Kyoto, Japan) supplemented with 10% heat inactivated foetal calf serum (Multiser, Trace Scientific Ltd., Melbourne, Australia), 1% chicken serum (Invitrogen, Carlsbad, CA, USA), and 50 μM 2-mercaptoethanol (Sigma, St.,Louis, MO, USA) at 39.5 °C, as described previously^42^. Mutations were introduced in the *ZNT2* cDNA and plasmids expressing ZNT2 and ZNT3 domain-swapped mutants were generated using two–step polymerase chain reaction (PCR) as described previously^24,38^. Domain-swapping sites were determined based on sequence alignment of ZNT2 and ZNT3 cytosolic His-rich loop regions, whereas N-terminal sites were selected based on sequences of YiiP proteins (Fig. 1). Multiple protein sequence alignment was generated using ClustalW. To establish cells stably expressing WT or mutant ZNT2 and ZNT3 proteins, DNA was electroporated as described previously^60^.

### Immunoblotting

Immunoblotting was performed as described previously^60^. Blotted polyvinylidene fluoride (PVDF) membranes (Millipore, Bedford, MA, USA) were blocked with 5% skimmed milk and 0.1% Tween-20 in phosphate-buffered saline and incubated with monoclonal anti-HA HA-11 (1:3000; BioLegend, San Diego, CA, USA, MMS-101P), monoclonal anti-FLAG M2 (1:3000; Sigma, F3165), anti-ZNT2 (1:3000)^24^, anti-Myc 9E10 (1:3000, Santa Cruz Biotechnology, Santa Cruz, CA, USA, sc-40), and anti-α-tubulin 12G10 (1:5000, deposited to the Developmental Studies Hybridoma Bank (DSHB) by Frankel, J. and Nelsen, E.M.) antibodies. For detection of immunoreactive bands, we used (at 1:3000 dilution) horseradish peroxidase-conjugated anti-mouse IgG antibody (GE Healthcare, Milwaukee, WI, USA, NA931). Fluorescence images were obtained using LAS 500 (GE Healthcare).

### Evaluation of WT and mutant ZNT2 and ZNT3 zinc transport function based on viability of znt1^−/−^mt^−/−^znt4^−/−^ cells in the presence of high zinc concentrations

The cells were seeded at a density of 10^5^ cells/ml in 96-well plates and treated with 50-90 µM ZnSO_4_ for 2 days as described previously^38^. The alamarBlue reagent (AbD Serotec, Ltd., Oxford, UK) was added to the culture media and the cells were incubated for 4 h. Absorbance was determined at 570 and 600 nm using PowerScan 4 (DS Pharma Biomedical, Osaka, Japan).

### Evaluation of the stability of ZNT2 mutants

The stability of mutant ZNT2 proteins was evaluated as described previously^24,42^. Cells expressing WT or mutant ZNT2 were treated with cycloheximide to block further protein synthesis and were collected periodically over 8 h. Total cell lysates were prepared and subjected to immunoblotting to monitor ZNT2 levels. The band intensity of each protein is the average of data from three independent experiments and is shown as the percentage of the intensity at 0 h (T_0_) after normalization. Densitometric quantification was performed using ImageQuant (GE Healthcare). Experiments were performed in triplicate.

### Statistical analyses

Data are represented as means ± standard deviation (SD) or standard error of mean (SEM). Statistical significance was determined using Dunnett’s test. *P*-values < 0.05 were considered statistically significant.

## Electronic supplementary material

Supplementary Information

## Data Availability

All data generated or analysed during this study are included in this published article (and its supplementary information files) or are available upon reasonable request.
