# Supplementary material for: Evaluation of the roles of the cytosolic N-terminus and His-rich loop of ZNT proteins using ZNT2 and ZNT3 chimeric mutants
Source: Sci Rep. 2018 Sep 20;8:14084. doi: 10.1038/s41598-018-32372-8 (PMC6147782; doi:10.1038/s41598-018-32372-8)
Supplement: Supplementary file 1 — Supplementary Information [file 41598_2018_32372_MOESM1_ESM.docx]

**Supplementary Information**

**Evaluation of the roles of the cytosolic N-terminus and His-rich loop of ZNT proteins using ZNT2 and ZNT3 chimeric mutants**

Short title: Dissecting biochemical properties of ZNT proteins

Kazuhisa Fukue^1^, Naoya Itsumura^1^, Natsuko Tsuji^1^, Katsutoshi Nishino^1^, Masaya Nagao^1^, Hiroshi Narita^2^ and Taiho Kambe^1^

^1^Division of Integrated Life Science, Graduate School of Biostudies, Kyoto University, Kyoto 606-8502, Japan

^2^Department of Food Science, Kyoto Women’s University, Kyoto 605-8501, Japan

**TABLE OF CONTENTS**

**Supplementary Table 1. Fifteen SLC30A2/ZNT2 SNPs investigated in the cell viability assay using znt1^-/-^mt^-/-^znt4^-/-^ cells.**

**Supplementary Figure 1. Evaluation of zinc transport function and protein stability of ZNT2 SNP mutants.**

**Supplementary Figure 2. Full-length immunoblot images used in Figure 2.**

**Supplementary Figure 3. Full-length immunoblot images used in Figure 3.**

**Supplementary Figure 4. Full-length immunoblot images used in Figure 4.**

**Supplementary Figure 5. Full-length immunoblot images used in Figure 5.**

**Supplementary Figure 6. Full-length immunoblot images used in Supplementary Figure 1.Supplementary Table 1. Fifteen *SLC30A2/ZNT2* SNPs investigated in the cell viability assay using *znt1^-/-^mt^-/-^znt4^-/-^* cells.**

| dbSNP ID | Nucleotide change | Amino acid change | Exon | gnomAD minor allele frequency^a^ |
| --- | --- | --- | --- | --- |
| rs781629255 | G>A | T20M | Exon 2 | 4.074e-6 |
| rs770977015 | C>A | L40F | Exon 2 | 1.625e-5 |
| rs746634287 | G>C | P65R | Exon 2 | 1.803e-5 |
| rs775287534 | C>A | M85I | Exon 2 | 1.082e-5 |
| rs745480516 | C>A | W122C | Exon 3 | 3.25e-5 |
| rs748793262 | C>T | R126Q | Exon 3 | 1.625e-5 |
| rs750998368 | T>C | H197R | Exon 5 | 4.064e-6 |
| rs759513690 | C>G | E213Q | Exon 5 | 5.279e-5 |
| rs774477332 | G>T | N214K | Exon 5 | 4.061e-6 |
| rs758261486 | C>T | E246K | Exon 6 | 4.072e-6 |
| rs746722376 | C>T | E279K | Exon 6 | 4.063e-6 |
| rs775726067 | G>T | A302D | Exon 7 | 6.097e-5 |
| rs771701593 | T>C | H307R | Exon 7 | 5.691e-5 |
| rs779238960 | C>T | V333M | Exon 8 | 8.168e-6 |
| rs753914698 | T>A | T336S | Exon 8 | 4.078e-6 |
| rs764331750 | T>G | S339R | Exon 8 | 4.074e-6 |
| rs761786738 | T>C | Q368R | Exon 8 | 2.846e-5 |

These SNPs were randomly selected from the dbSNP database (http://www.ncbi.nlm.nih.gov/snp); all are minor alleles.

^a^ genome Aggregation Database (gnomAD), containing 123136 exome sequencing data and 15486 whole-genome sequencing data from unrelated individuals from different populations.

**Supplementary Figure 1. Evaluation of zinc transport function and protein stability of ZNT2 SNP mutants.** (***A***) Expressing ZNT2_(E279K)_ in *znt1^-/-^mt^-/-^znt4^-/-^* cells failed to confer resistance to high zinc concentrations, similar to expressing TNZD-causing mutant ZNT2_(G280R)_. (***B***) Evaluation of the protein stability of E279K ZNT2 mutant. Expression levels of WT, ZNT2_(E279K)_ and ZNT2_(G280R)_ proteins at each time point are shown, with representative results of immunoblotting depicted in the lower panel. Data are represented as the means ± SEM of triplicate experiments (lower sub-panels). Asterisk (*) denotes a significant difference between WT and mutant ZNT2 protein levels (*P* <0.05 by Dunnett's test). Tubulin was used as the loading control. (***C***) Human ZNT2 protein sequences (residues 127 - 176 and 250 - 299) aligned to *E. coli* and *S. oneidensis* YiiP. Identical and similar amino acids are indicated by * and +, respectively. ZNT2 Glu 279 residue is highlighted (red) and acidic and basic amino acid residues contributing to the formation of salt-bridges are highlighted orange and cyan, respectively.

**Supplementary Figure 2. Full-length immunoblot images used in Figure 2.** (*A*) The panel used for Fig. 2A is boxed. (*B*) The panel used for Fig. 2B is boxed. (*C*) The panel used for Fig. 2C is boxed. (*D*) The panel used for Fig. 2D is boxed. (*E*) The panel used for Fig. 2E is boxed. (*F*) The panel used for Fig. 2F is boxed. The same blot was used sequentially (after stripping) for detection of HA- or FLAG-tagged ZNT2 and Tubulin in this order. The position of the protein size marker is indicated on the *right*.

**Supplementary Figure 3. Full-length immunoblot images used in Figure 3.** (*A*) The panel used for Fig. 3A is boxed. (*B*) The panel used for Fig. 3B is boxed. (*C*) The panel used for Fig. 3C is boxed. (*D*) The panel used for Fig. 3D is boxed. (*E*) The panel used for Fig. 3E is boxed. (*F*) The panel used for Fig. 3F is boxed. (*G*) The panel used for Fig. 3G is boxed. The same blot was used sequentially (after stripping) for detection of HA-, FLAG- or Myc-tagged ZNT2 and Tubulin in this order. The position of the protein size marker is indicated on the *right*.

**Supplementary Figure 4. Full-length immunoblot images used in Figure 4.** (*A*) The panel used for Fig. 4A is boxed. (*B*) The panel used for Fig. 4B is boxed. The same blot was used sequentially (after stripping) for detection of HA-, Myc- or FLAG-tagged ZNT3 and Tubulin in this order. The position of the protein size marker is indicated on the *right*.

**Supplementary Figure 5. Full-length immunoblot images used in Figure 5.** (*A*) The panel used for Fig. 5A is boxed. (*B*) The panel used for Fig. 5B is boxed. (*C*) The panel used for Fig. 5C is boxed. In *(A)* and *(B)*, the same blot was used sequentially (after stripping) for detection of HA- or FLAG-tagged ZNT2 and Tubulin in this order. In *(C)*, the blot was cut into halves, and each was used for detection of HA-tagged ZNT2 and Tubulin. The position of the protein size marker is indicated on the *right.*

**

**Supplementary Figure 6. Full-length immunoblot images used in Supplementary Figure 1.** (*A*) The panel used for Supplementary Fig. 1A is boxed. (*B*) The panel used for Supplementary Fig. 1B is boxed. In *(A)*, the same blot was used sequentially (after stripping) for detection of HA-tagged ZNT2 and Tubulin in this order. In *(B)*, the blot was cut into halves, and each was used for detection of HA-tagged ZNT2 and Tubulin. The position of the protein size marker is indicated on the *right.*
